# Optimized Rapeseed Oils Rich in Endogenous Micronutrients Protect High Fat Diet Fed Rats from Hepatic Lipid Accumulation and Oxidative Stress

**DOI:** 10.3390/nu7105407

**Published:** 2015-10-14

**Authors:** Jiqu Xu, Xiaoli Liu, Hui Gao, Chang Chen, Qianchun Deng, Qingde Huang, Zhonghua Ma, Fenghong Huang

**Affiliations:** 1 Department of Product Processing and Nutriology, Oil Crops Research Institute, Chinese Academy of Agricultural Sciences, 2 Xudong Second Road, Wuhan 430062, China; xujiqu@foxmail.com (J.X.); liuxiaoli_ocri@sina.com (X.L.); qianch2@foxmail.com (Q.D.); viperson1973@foxmail.com (Q.H.); 2 Hubei Key Laboratory of Lipid Chemistry and Nutrition, Oil Crops Research Institute, Chinese Academy of Agricultural Sciences, 2 Xudong Second Road, Wuhan 430062, China; 3 Department of Nutrition and Food Hygiene, School of Public Health, Tongji Medical College, Huazhong University of Science and Technology, 13 Hangkong Road, Wuhan 430030, China; gaohuitj@yeah.net; 4 Department of Gastroenterology, The First People’s Hospital of Yichang, The People’s Hospital of China Three Gorges University, 2 Jiefang Road, Yichang 443000, China; chenchang1976@163.com; 5 Department of Gastroenterology, The People’s Hospital of China Three Gorges University, 2 Jiefang Road, Yichang 443000, China; 6 Functional Oil Laboratory Associated by Oil Crops Research Institute, Chinese Academy of Agricultural Sciences and Infinite (China) Co., LTD., 66 Jianzhong Road, Guangzhou 510665, China; zhhuama@163.com

**Keywords:** optimized rapeseed oils, micronutrients, lipid accumulation, oxidant stress, nonalcoholic fatty liver disease

## Abstract

Micronutrients in rapeseed exert a potential benefit to hepatoprotection, but most of them are lost during the conventional refining processing. Thus some processing technologies have been optimized to improve micronutrient retention in oil. The aim of this study is to assess whether optimized rapeseed oils (OROs) have positive effects on hepatic lipid accumulation and oxidative stress induced by a high-fat diet. Methods: Rats received experiment diets containing 20% fat and refined rapeseed oil or OROs obtained with various processing technologies as lipid source. After 10 weeks of treatment, liver was assayed for lipid accumulation and oxidative stress. Results: All OROs reduced hepatic triglyceride contents. Microwave pretreatment-cold pressing oil (MPCPO) which had the highest micronutrients contents also reduced hepatic cholesterol level. MPCPO significantly decreased hepatic sterol regulatory element-binding transcription factor 1 (SREBP1) but increased peroxisome proliferator activated receptor α (PPARα) expressions, and as a result, MPCPO significantly suppressed acetyl CoA carboxylase and induced carnitine palmitoyl transferase-1 and acyl CoA oxidase expression. Hepatic catalase (CAT) and glutathione peroxidase (GPx) activities as well as reduced glutathione (GSH) contents remarkably increased and lipid peroxidation levels decreased in parallel with the increase of micronutrients. Conclusion: OROs had the ability to reduce excessive hepatic fat accumulation and oxidative stress, which indicated that OROs might contribute to ameliorating nonalcoholic fatty liver induced by high-fat diet.

## 1. Introduction

Concurrent with rising obesity prevalence, nonalcoholic fatty liver disease (NAFLD) has emerged as the most frequent chronic liver disease worldwide. It is estimated to affect approximately 20%–30% general population in Western [1] and other developing countries [2]. High-fat intake is the most common single cause for NAFLD [3] since long-term consumption of this type of diet promotes obesity and the development of the metabolic syndrome which are highly associated with NAFLD [4]. This disease represents a wide spectrum of diseases ranging from simple steatosis to nonalcoholic steato hepatitis (NASH) to advanced fibrosis and cirrhosis. Simple steatosis is considered relatively benign and has the best prognosis, but NASH is aggressive which may lead to progressive liver disease. Therefore, the transition from simple steatosis to NASH is a key issue in the NAFLD field. Although the etiology of this disease has remained elusive, oxidative stress and excessive lipid accumulation are two requisites for NAFLD progression [5].

Rapeseed oil is the third most important vegetable oil after palm and soybean oils in the world. It has the lowest concentration of saturated fatty acids of all common edible oils and predominant monounsaturated fatty acids. In addition, this oil is the main source of the α-linolenic acid (ALA) as well as a well-balanced ratio between ALA and linoleic acid [6]. The native composition of fatty acids of rapeseed oil has been found to induce many physiological responses in inflammatory cytokines, carbohydrate metabolism, adipose tissue, and adipokines [7], which make this oil of benefit to health. 

In addition to triacylglycerols, rapeseed oil contains many health-related compounds such as phenolic compounds, tocopherols, and phytosterols which have been recognized to possess various health benefits. These micronutrients have been recognized to play a positive role in various health benefits. For example, phenolic compounds and tocopherols have excellent antioxidant activity due to their redox properties and their function as potential chelators of prooxidant metals or free radical scavengers. Phenolics are able to improve hepatic lipid accumulation caused by high-fat diet [8] but phytosterols effectively decrease hepatic cholesterol level by inhibiting cholesterol absorption [9]. All these beneficial effects make these bioactive components a very important contribution to the recovery from or prevention of liver damage. However, since these micronutrients originate from the rapeseed seed, the traditional processing technology (extraction and refining) currently used in the world leads to substantial losses of these bioactive components. Therefore some technologies in oilseed processing have been optimized to enhance micronutrient extraction and minimal refining for the improvement of micronutrient retention in oil. The present study was designed to investigate whether the various endogenous micronutrient-enriched optimized rapeseed oils (OROs) are capable of decreasing hepatic lipid accumulation and oxidative stress in rats fed a high-fat diet.

## 2. Experimental Section

### 2.1. Oils Preparation

The refined rapeseed oil (RRO) was produced with conventional extraction and refining processing technology. Three OROs with higher contents of micronutrients (CPO, DCPO, and MPCPO) were obtained by adopting different technical procedures: cold pressing (CP), dehulling-cold pressing (DCP), and microwave pretreatment-cold pressing (MPCP). The endogenous micronutrients and fatty acids compositions in different oils were presented in Table 1 and Table 2, respectively.

**Table 1:** Endogenous micronutrients in different rapeseed oils.

mg/kg Oil	RRO	CPO	DCPO	MPCPO
Phenols (in equivalent, sinapic acid)	8	34	43	645
Phytosterols	9592	9902	8834	11,027
Tocopherol	461	541	594	600
Phospholipids	22	600	590	1330

RRO: the refined rapeseed oil group; CPO: cold pressing rapeseed oil group; DCPO: dehulling-cold pressing rapeseed oil group; MPCPO: microwave pretreatment-cold pressing rapeseed oil group.

**Table 2:** Major fatty acid compositions in different rapeseed oils.

Fatty Acid (Weight %)	RRO	CPO	DCPO	MPCPO
C16:0	2.413	2.500	2.485	2.440
C18:0	3.667	3.769	3.846	3.548
C18:1	66.19	66.075	66.287	66.561
C18:2	16.822	16.798	17.088	16.447
C18:3	9.146	9.281	8.463	9.431

RRO: the refined rapeseed oil group; CPO: cold pressing rapeseed oil group; DCPO: dehulling-cold pressing rapeseed oil group; MPCPO: microwave pretreatment-cold pressing rapeseed oil group.

### 2.2. Animals and Diets

Forty adult male rats of Wister strain weighing 160 ± 10 g were obtained from Vital River Laboratory Animal Center (Beijing, China). The animals were housed individually in polypropylene cages and maintained at a controlled ambient temperature (24 ± 1 °C) under diurnal conditions (light-dark: 08:00–20:00) with access to laboratory chow and tap water ad libitum. After acclimating for seven days in this laboratory environment, rats were randomly divided into four groups (*n* = 10) according to the type of oils: RRO (control group), CPO, DCPO, and MPCPO groups. All animals were fed purified high-fat diet for 10 weeks which containing 20% casein, 35% maize starch, 15% glucose, 5% cellulose, 3.5% mineral mixture (AIN-93M), 1% vitamin mixture (AIN-93M), 0.2% choline bitartrate, 0.3% dl-methionine, and 20% fat. The fat in the diet was the four different rapeseed oils mentioned above. All animals were weighed twice a week and food intake was measured weekly. The animals were cared for in accordance with *the Guiding Principles in the Care and Use of Animals* [10]. The experiment was approved by the Oil Crops Research Institute Council on Animal Care Committee, Chinese Academy of Agricultural Sciences (License Number 004/2014).

### 2.3. Tissue Preparation

Animals were fasted for 16 h before killed under anaesthesia. The livers were harvested immediately. A small piece of the same site of the right liver lobe from each rat was fixed with 4% paraformaldehyde for histologic analysis and the remaining liver tissue was snap frozen in liquid nitrogen and stored at −80 °C until analysis.

### 2.4. Lipid Content

Liver lipids were extracted with chloroform/methanol (2:1, v/v) according to the method described by Folch [11]. Total triglyceride (TG) and total cholesterol (TC) levels were determined with commercial kits (Zhongsheng Beikong Biotech Company, Beijing, China).

### 2.5. Antioxidant Capacity and Lipid Peroxidation

Liver was weighed and a 10% homogenate was prepared in a 50 mmol/L phosphate buffer (pH 7.0) containing 0.1 mmol/L EDTA. The homogenate was centrifuged at 3500 rpm for 10 min at 4 °C for superoxide dismutase (SOD), catalase (CAT), glutathione peroxidase (GPx), reduced glutathione (GSH), and thiobarbituric acid reactive substances (TBARS) tests.

#### 2.5.1. Determination of SOD

SOD activity was measured basing on the method of Kono [12] with slight modification. Briefly, the reaction was initiated by mixing an aliquot of homogenate supernatant with 0.5 mM hypoxanthine, 0.5 mM hydroxylamine and 0.01 U xanthine oxidase in the buffer, containing of 104 mM potassium phosphate, 78 mM sodium borate, and 0.025 mM EDTA (PH 7.0) at 37 °C for 30 min in a reaction volume of 100 μL. The reaction was terminated by adding 0.2 mL of 16% (v/v) acetic acid solution containing 2.6 mM sulfanilic acid and 38.6 μM naphthyl ethylenediamine and the absorbance at 550 nm was recorded for the calculation of SOD activity. Under the conditions, one nitroso unit (NU) of enzyme activity was calculated as that inhibiting 50% of the oxidation of hydroxylamine without an enzyme source.

#### 2.5.2. Determination of GPx

GPx activity was assayed by the method of Sazuka [13] with slight modification. Briefly, the homogenate supernatant mixed with GSH and hydrogen peroxide was incubated at 37 °C for 3 min, followed by the addition of 10% trichloro acetic acid (TCA). After centrifugation, the supernatant was collected and mixed with disodium hydrogen phosphate and 5,5,-dithiobis(2-nitro-benzoic acid) (DTNB). The absorbance was recorded at 412 nm. The unit of GPx activity was expressed as micromoles GSH oxidation per min per mg protein.

#### 2.5.3. Determination of CAT

CAT activity was determined according to the method of Goth [14] with slight modification. Briefly, 50 μL of sample was mixed with 50 μL of substrate (6.5 μM hydrogen peroxide in phosphate buffer) for 60 s, then 100 μL of 32.4 mM ammonium molybdate solution were added and absorbance change was measured at 405 nm. One unit of the enzyme was defined as millimoles of hydrogen peroxide degraded per min per mg protein.

#### 2.5.4. Determination of GSH

The GSH content was calculated by the method of Moron [15] with slight modification. Briefly, the protein in sample was precipitated with 50% TCA and then centrifuged at 1000 g for 5 min. The reaction mixture containing 50 μL of supernatant, 200 μL of 0.2 M Tris–EDTA buffer (PH 8.9) and 10 μL of 0.01 M 5,5,-dithiobis (2-nitro-benzoic acid) (DTNB) was kept at room temperature for 5 min, and then measured at 412 nm. The GSH concentration was calculated using a GSH standard curve.

#### 2.5.5. Determination of TBARS

TBARS level was measured by the method of Buege and Aust [16]. Briefly, the homogenate supernatant was incubated reagent containing 0.375% thiobarbituric acid (TBA), 15% TCA, 0.25 M HCl, and 6.8 mM 2,6-di-tert-butyl-4-methylphenol (BHT) for 60 min in a boiling water bath. The mixture was centrifuged at 3000 rpm for 15 min, the absorbance of the supernatant was recorded at 532 nm by using 1,1,3,3-tetraethoxypropane (TEP) as standard. The lipid peroxidation was expressed as TBARS in nanomoles per mg protein.

#### 2.5.6. Assay of Protein Concentration

The protein concentration was determined according to the method of Lowry [17], using bovine serum albumin (BSA) as standard.

### 2.6. Western Blot Analysis

Liver samples were lysed in ice-cold RIPA buffer supplemented with 1 mM PMSF (Sigma, St. Louis, MO, USA) and then left at 4 °C for 2 h. The supernatant was collected by centrifuge at 10,000 g for 15 min at 4 °C. For SDS–PAGE, samples were mixed with SDS sample buffer and incubated at 98 °C for 5 min. Western blot was performed with the following antibodies: β-actin mAb (#3700, Cell Signaling, Danvers, MA, USA), peroxisome proliferator activated receptor α (PPARα) pAb (ab8934, Abcam, Cambridge, UK), sterol regulatory element-binding transcription factor 1 (SREBP1) mAb (sc-365513, Santa Cruz, Santa Cruz, CA, USA), and 3-hydroxy-3-methylglutaryl-CoA reductase (HMGCR) mAb (ab174830, Abcam, Cambridge, UK). 

### 2.7. Quantitative Real-Time PCR

Total RNA of the liver was isolated using Trizol^TM^ (Invitrogen, Carlsbad, CA, USA) as directed by the manufacturer’s instruction of the kit. Using the RT system (Takara Bio, Dalian, China), cDNA was synthesized from total RNA. Real-time polymerase chain reaction (PCR) was performed with SYBR^®^ Premix Ex Taq^TM^ II (Takara Bio, Dalian, China), using the ABI 7900HT real-time thermocycler (Applied Biosystems, Forster, CA, USA). The dissociation curve of each gene was performed and analyzed using the ABI 7900HT software, and the result confirmed the product specificity. Each sample was analyzed three times and normalized to β-actin. The results of real-time PCR were analyzed with the 2^−ΔΔCt^ method. The sequences of the primers for the genes in this study were as follows. fatty acid synthase (FAS): forward 5′-GGACATGGTCACAGACGATGAC-3′, reverse 5′-GTCGAACTTGGACAGATCCTTCA-3′; acetyl CoA carboxylase (ACC): forward 5′-GCCTCTTCCTGACAAACGAG-3′, reverse 5′- TCCATACGCCTGAAACATGA-3′; carnitine palmitoyl transferase-1 (CPT-1): forward 5′-AACTTTGTGCAGGCCATGATG-3′, reverse 5′-AGCTTGTGAGAAGCACCAGCA-3′; acyl CoA oxidase (ACO): forward 5′-ATCTCTGTGGTTGCTGTGGAGTCA-3′, reverse 5′- TCTGGATGCTTCCTTCTCCAAGGT-3′.

### 2.8. Statistical Analyses

Results were expressed as mean ±standard error of the mean (SEM). Statistical analysis were based on one-way analysis of variance (ANOVA), followed by the Fisher PLSD post hoc test if the overall differences were significant. All statistical analyses were performed using SPSS 13.0 statistical software (SPSS Inc., Chicago, IL, USA) and the limit of statistical significance was set at *p* < 0.05.

## 3. Results

### 3.1. Liver Morphology

**Figure 1:**
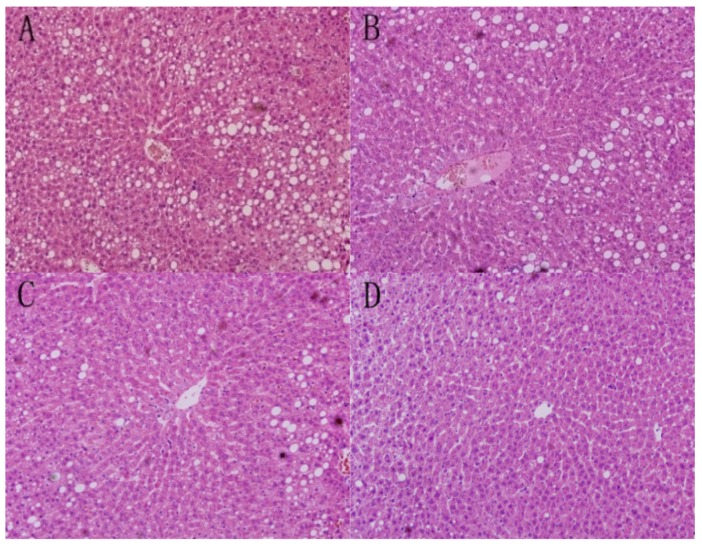
Liver sections stained with haematoxylin and eosin (original magnification ×200) from each group: (**A**) the refined rapeseed oil group; (**B**) cold pressing rapeseed oil group; (**C**) dehulling-cold pressing rapeseed oil group; and (**D**) microwave pretreatment-cold pressing rapeseed oil group.

As shown in Figure 1, liver morphology of RRO group revealed extensive macrovesicular steatosis and scattered foci of microvesicular steatosis. The circular lipid droplets in both size and number were substantially reduced in the liver of all the three optimised groups and the rats in MPCPO group had minimal fatty changes in hepatocytes. No evidence of inflammation was found in any experimental group.

### 3.2. Liver Lipids Contents

**Figure 2:**
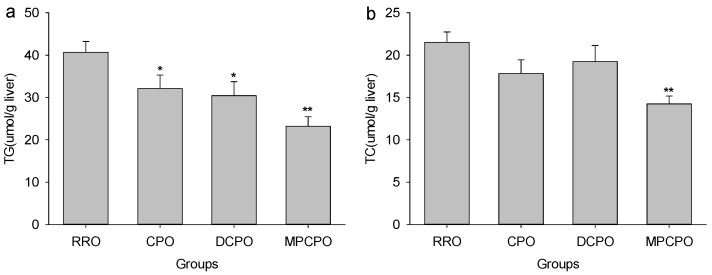
Effects of optimized rapeseed oils (OROs) on (a) hepatic triglyceride (TG) and (b) cholesterol (TC) contents of rats fed a high-fat diet. RRO: the refined rapeseed oil group; CPO: cold pressing rapeseed oil group; DCPO: dehulling-cold pressing rapeseed oil group; MPCPO: microwave pretreatment-cold pressing rapeseed oil group. Bars represent the mean ± standard error of the mean (SEM) from 10 animals in each group. * *p* < 0.05 and ** *p* < 0.01 compared to the RRO group.

The liver contents of TG and TC in different rapeseed oils-treated rats are presented in Figure 2. All the three OROs had the abilities to lower the liver TG levels significantly but MPCPO also noticeably decrease hepatic cholesterol level when compared with RRO.

### 3.3. Liver Proteins Expressions

**Figure 3:**
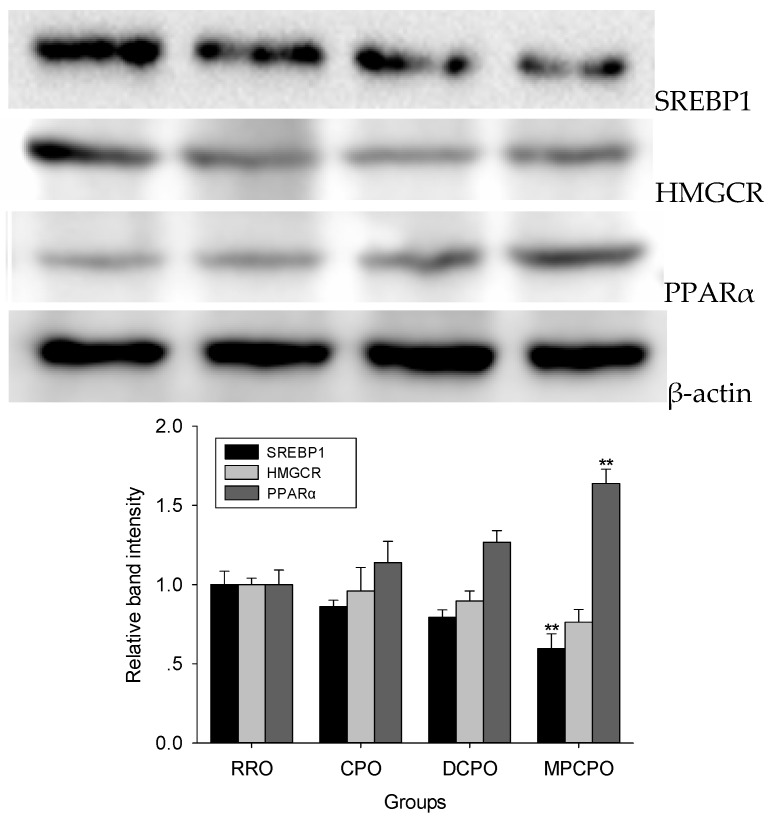
Effects of optimized rapeseed oils (OROs) on hepatic lipids synthesis related proteins expression. Representative immunoblot comparing relative levels of sterol regulatory element-binding transcription factor 1 (SREBP1), 3-hydroxy-3-methylglutaryl-CoA reductase (HMGCR), and peroxisome proliferator activated receptor α (PPARα) proteins in liver are shown above graph. RRO: the refined rapeseed oil group; CPO: cold pressing rapeseed oil group; DCPO: dehulling-cold pressing rapeseed oil group; MPCPO: microwave pretreatment-cold pressing rapeseed oil group. Bars represent the mean ± standard error of the mean (SEM) from four independent experiments. ** *p* < 0.01 compared to the RRO group.

SREBP1 is a key transcription factor responsible for fatty acids biosynthesis. As shown in Figure 3, when compared with the RRO group, the expression of HMGCR showed decreasing trends in all OROs groups, whereas the significantly lower expression of SREBP1 was observed in MPCPO group. The expression of PPARα in MPCPO group was substantially increased than that in RRO group.

### 3.4. Liver mRNA Expressions

**Figure 4:**
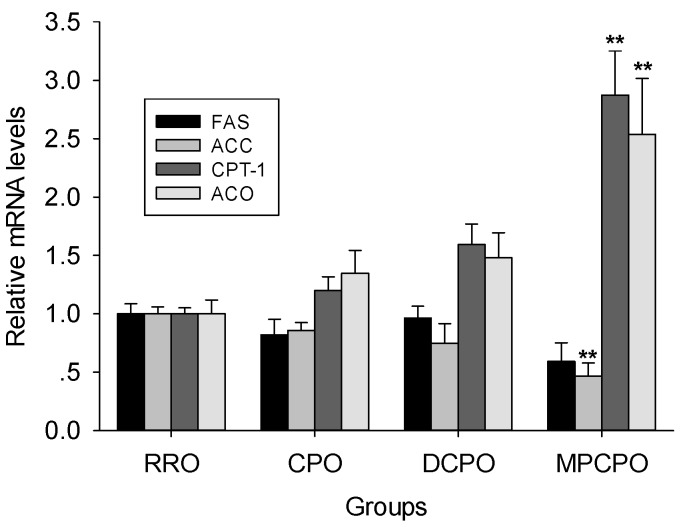
Effects of optimized rapeseed oils (OROs) on hepatic mRNA levels of fatty acid synthase (FAS), acetyl CoA carboxylase (ACC), carnitine palmitoyl transferase-1 (CPT-1), and acyl CoA oxidase (ACO). RRO: the refined rapeseed oil group; CPO: cold pressing rapeseed oil group; DCPO: dehulling-cold pressing rapeseed oil group; MPCPO: microwave pretreatment-cold pressing rapeseed oil group. Bars represent the mean ± standard error of the mean (SEM) from four independent experiments. ** *p* < 0.01 compared to the RRO group.

FAS and ACC, two important SREBP-1 target enzymes [18], are essential for fatty acid synthesis. Although the trend to decrease of FAS mRNA expression did not achieve the statistical significance in all OROs groups, the mRNA expression of ACC in MPCPO group remarkably lower than that of RRO group (Figure 4). Conversely, MPCPO significantly increased the mRNA expression of CPT-1 and ACO which are PPAR α-induced rate-limiting enzymes of fatty acid oxidation [19,20].

### 3.5. Liver Antioxidative Capacity and Lipid Peroxidation

**Figure 5:**
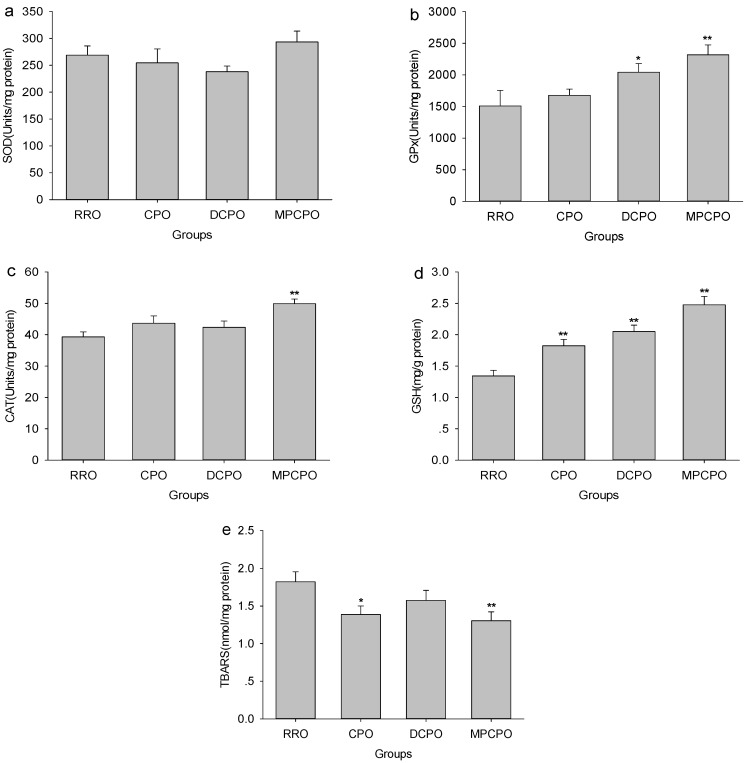
Effects of optimized rapeseed oils (OROs) on hepatic superoxide dismutase (SOD) (a), glutathione peroxidase (GPx) (b) and catalase (CAT) (d) activities as well as glutathione (GSH) (d) and thiobarbituric acid reactive substances (TBARS) (e) contents in rats fed a high-fat diet. RRO: the refined rapeseed oil group; CPO: cold pressing rapeseed oil group; DCPO: dehulling-cold pressing rapeseed oil group; MPCPO: microwave pretreatment-cold pressing rapeseed oil group. Bars represent the mean ± standard error of the mean (SEM) from 10 animals in each group. * *p* < 0.05 and ** *p* < 0.01 compared to the RRO group.

With the aim of determining if the endogenous bioactive compounds reflect antioxidative capacity, the effects of OROs treatment on several primary antioxidant defense components were measured. As can be seen in Figure 5, although the RRO and the three optimized groups had comparable SOD activities, the GPx activities in DCPO and MPCPO groups and the CAT activity in MPCPO group were statistically higher than that in RRO group. The GSH levels elevated substantially with the contents of micronutrients in oils and the highest level was detected in the MPCPO group. When TBARS were determined as the biomarkers of lipid peroxidation, significant declines in hepatic TBARS contents were observed in CPO and MPCPO groups.

## 4. Discussion

The optimum fatty acids composition presented in rapeseed oil makes this kind of oil competent to exert many health effects [21,22,23]. For example, replacing dairy fat with rapeseed oil led to rapid and clinically significant reductions in TG, low-density lipoprotein cholesterol (LDL-C) [21]. Unfortunately, oleic acid which is predominant in rapeseed oil is oxidized at a remarkably fast rate [24,25] but has a greater uptake rate [24], the combined effects make some passive effects on liver with the treatment of rapeseed oil, as shown by significant hepatic lipid accumulation and liver dysfunction which are similar to that of lard even though the diets were not high in fat [26]. This fact evokes the concern for adverse hepatic effects alongside of consumption of rapeseed oil. Many kinds of micronutrients present in rapeseed oil have been reported to have excellent bioactivity. All these bioactive compounds can work synergistically to regulate biological functions. In our previous study, artificially adding these micronutrients to refined rapeseed oil exerted an unambiguous restraining effect on liver damage caused by high-fat diet [27]. Therefore OROs with high content of bioactive compounds may be competent for the similar pattern of hepatoprotection.

Unsurprisingly, significant steatosis in liver was detected when challenged with RRO in the present study, which suggested that RRO resulted in the pronounced fat accumulation. All the OROs, especially MPCPO, retard the development of hepatic steatosis substantially. Consistent with the liver morphology, the OROs can efficiently reduce hepatic TG contents. Rapeseed is rich in phenolic compounds including: sinapic, salicylic, protocatechuic, *p*-hydroxybenzoic, gentisic, ferulic, *p*-coumaric, cinnamic, caffeic, and syringic acids, which are all presented in the OROs. Those phenolics are capable of reducing fatty acid synthesis by suppressing numerous lipogenic enzymes, such as FAS and ACC as well as lipogenic transcriptional factor SREBP1 [28,29,30], and promote hepatic lipid oxidation by stimulating the expression of PPARα [28,29]. The accumulation of cholesterol is also associated with the pathogenesis of NAFLD [31,32]. Recent evidence suggested that those phenolics can inhibit the protein expression of HMGCR which is the rate-limiting enzyme in cholesterol biosynthesis, and sterol regulatory element-binding protein-2 (SREBP-2) which targets the enzymes for cholesterogenesis. Therefore, hepatic cholesterol synthesis was inhibited by phenolics [28]. Phytosterols have similar structures to cholesterol but are poorly absorbed [9]. They interfere intestinal cholesterol absorption including recirculating endogenous biliary cholesterol which is a crucial step in cholesterol elimination [9]. These further suggested that even though little cholesterol is presented in the rodent diet, interference of cholesterol absorption was still the primary mechanism for the cholesterol-lowering effect of phytosterols. Therefore, although these compounds may increase the activity of HMGCR in liver [33], phytosterols still lower hepatic cholesterol content notably [34,35]. Besides, phytosterols can promote fecal fatty acid loss, thus correlating with plasma and hepatic TG reduction [34]. Another micronutrient tocopherol also had strong effects on TG-lowering response in NAFLD, and the mechanisms are at least linked to increased hepatic PPAR-α expression and/or decreased PPAR-γ expression [36,37]. In the present study, the OROs, especially MPCPO, decreased the expression of SREBP1 and HMGCR but increased the expression of PPARα in liver. As results, MPCPO significantly suppressed ACC expression and potently induced CPT-1 and ACO expression. These presented evidences that the OROs, especially MPCPO, could decrease hepatic triglyceride and cholesterol contents by suppressing hepatic lipogenesis and cholesterogenesis as well as promoting lipid oxidation.

High-fat diet impairs hepatic antioxidant capacity, which results in remarkable oxidative stress [36,38]. Phenolic compounds have pronounced antioxidant capacity [39,40] which is accomplished by not only chelating with transition metal ions and scavenging reactive oxygen indirectly, but also induction of antioxidant enzymes and inhibition of prooxidant enzymes indirectly [41,42,43]. For instance, phenolic compounds enhance the activities of antioxidant enzymes CAT, GPx, and SOD and increase the level of GSH in liver [41,42,43]. As a kind of potent lipid-soluble antioxidants, tocopherols are well-known to have potent effects on slashing liver lipoperoxidation induced by a high-fat diet [36]. In addition to their cholesterol-lowering properties, phytosterols also increase some antioxidant enzymes activities such as GPx, CAT, and SOD as well as some nonenzymatic antioxidants such as vitamin C, vitamin E, and GSH in liver [44,45]. The synergistic combination of these antioxidants offers significantly higher antioxidant capacity than provided by each separate compound [46,47,48]. In the present study, optimized rapeseed oil, especially MPCP, triggered the increased hepatic activities of antioxidant enzymes (GPx and CAT) and content of GSH as well as consistent reduced hepatic TBARS levels, which showed evidence for elevated antioxidant capacity as well as attenuated oxidative stress and thus meant the prevention of progression of fatty livers. Consistent with these observations, ours and others’ previous research also reported that optimized oils or these micronutrients fortified oils are able to increase antioxidant activities and decrease lipid peroxidation in liver as well as in brain and plasma [27,49,50,51,52].

Collectively, since OROs naturally contained high contents of micronutrients, the ability to improve hepatic lipid contents, coupled with their ability to reduce oxidative stress in liver, favored their use as functional food in the prevention of NAFLD. 
